# Spin-selected electron transfer in liquid–solid contact electrification

**DOI:** 10.1038/s41467-022-32984-9

**Published:** 2022-09-05

**Authors:** Shiquan Lin, Laipan Zhu, Zhen Tang, Zhong Lin Wang

**Affiliations:** 1 Beijing Institute of Nanoenergy and Nanosystems, Chinese Academy of Sciences, Beijing, 100083 P. R. China; 2 School of Nanoscience and Technology, University of Chinese Academy of Sciences, Beijing, 100049 P. R. China; 3 School of Materials Science and Engineering, Georgia Institute of Technology, Atlanta, GA 30332−0245 USA

**Keywords:** Electronic properties and materials, Spintronics, Surfaces, interfaces and thin films, Magnetic properties and materials, Chemical physics

## Abstract

Electron transfer has been proven the dominant charge carrier during contact electrification at the liquid–solid interface. However, the effect of electron spin in contact electrification remains to be investigated. This study examines the charge transfer between different liquids and ferrimagnetic solids in a magnetic field, focusing on the contribution of O_2_ molecules to the liquid–solid contact electrification. The findings reveal that magnetic fields promote electron transfer at the O_2_-containing liquid–solid interfaces. Moreover, magnetic field-induced electron transfer increases at higher O_2_ concentrations in the liquids and decreases at elevated temperatures. The results indicate spin-selected electron transfer at liquid–solid interface. External magnetic fields can modulate the spin conversion of the radical pairs at the O_2_-containing liquid and ferrimagnetic solid interfaces due to the Zeeman interaction, promoting electron transfer. A spin-selected electron transfer model for liquid–solid contact electrification is further proposed based on the radical pair mechanism, in which the HO_2_ molecules and the free unpaired electrons from the ferrimagnetic solids are considered radical pairs. The spin conversion of the [HO_2_• •e^−^] pairs is affected by magnetic fields, rendering the electron transfer magnetic field-sensitive.

## Abstract (web summary)

Electron transfer has been shown to contribute to contact electrification at liquid–solid interface. Here, authors investigate the magnetic field effect on the liquid–solid electron transfer and propose a spin conversion model for the liquid–solid contact electrification.

## Introduction

Contact electrification (CE) has been known since ancient Greek times. The mechanisms of the solid-solid CE have been widely discussed and different theories have been well established, such as electron transfer^1–4^, ion transfer^5,6^ and material transfer^7–11^. While the identity of charge carriers (electron or ion transfer) for liquid–solid cases has been debated for decades without a conclusive resolution. However, it was recently proposed that electron transfer plays a dominant role in liquid–solid CE^12–14^, providing an insight into electric-double layer (EDL) formation and related fields, such as mechanochemistry, electrocatalysis, electrochemical storage, electrophoresis, and liquid–solid triboelectric nanogenerators (L-S TENGs)^15^. As an important intrinsic property of electrons, the spin should be considered during liquid–solid CE since electron transfer may contribute significantly to this process. It is widely accepted that electron transfer between two species is spin-conservative and follows the Pauli exclusion principle^16–20^. Therefore, liquid–solid CE is expected to be spin-dependent. The spin direction of the transferred electrons must be parallel to that of the acceptor states at the interface for electron transfer to occur. However, this expectation has not been verified experimentally.

The radical pair mechanism (RPM) introduced in the 1960s, considers spin-selected electron transfer during chemical reactions^21–23^. The spin configurations of the radical pairs with unpaired electrons can be modulated by an external magnetic field via Zeeman interaction, further affecting spin-selected electron transfer during chemical reactions^24–26^. Based on the RPM, magnetic field sensitive spin-selected electron transfer may theoretically occur during CE between water and ferrimagnets, which display a spin-split band structure^27^. Electrons are naturally spin-polarized at the Fermi level of ferrimagnets, suggesting the presence of a large number of unpaired electrons on the ferrimagnet surface^28^. For the liquid side, the ground spin state of H_2_O molecule is singlet with all paired electrons, making the H_2_O molecule antimagnetic. However, the oxygen (O_2_) molecules dissolved in water are in a triplet ground state^29^. The frontier π^*^ orbitals of the ground state O_2_ molecules are occupied by two unpaired electrons in parallel alignment^30^. Studies have recently demonstrated that the oxygen evolution reaction (OER) and oxygen reduction reaction (ORR) activity can be sensitive to external magnetic field^30–33^, suggesting spin-selected electron transfer involving O_2_ molecules, which is successfully explained by RPM^34–36^. Therefore, spin-selected electron transfer during liquid–solid CE can be verified by exposing the liquid–solid interface to a magnetic field. During CE between water and solids, the electron transfer may occur between two free oxygenous radicals at the interface, which can be considered as a radical pair due to the existence of the unpaired electrons. The magnetic field may affect the spin conversion of the radical pairs due to the Zeeman interaction, promoting electron transfer during liquid–solid CE. As alternative charge carrier candidates during liquid–solid CE, OH^−^ and H_3_O^+^ are both in a singlet ground state and display an exceedingly weak response to magnetic fields. Therefore, spin-selected electron transfer contributes to liquid–solid CE if the latter is sensitive to magnetic fields. Moreover, this provides a strategy for controlling the EDL structure by applying a magnetic field, with broad implications in EDL-related fields.

This study examines the CE between different liquids and ferrimagnetic solids and measures the transferred charge density using dual harmonic Kelvin probe force microscopy (DH-KPFM), which can be used in a liquid environment^37,38^. The paper focuses on the effect of external magnetic fields on the CE between liquids and solids. According to the results, the dissolved O_2_ molecules in the liquid contribute to liquid–solid CE. Moreover, the magnetic field can promote charge transfer between the O_2_-containing liquids and the ferrimagnetic samples, suggesting that spin-selected electron transfer occurs during liquid–solid CE. An electron transfer model considering electron spin for liquid–solid CE is proposed based on RPM theory, providing a perspective for understanding magnetic field-controlled chemical reactions.

## Results

### Effect of the magnetic field on the liquid–solid CE

**Fig. 1:**
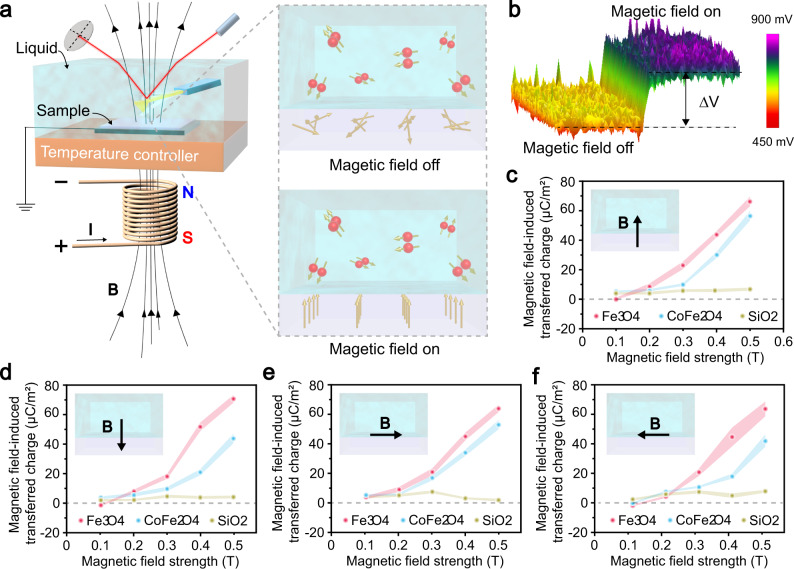
The effect of the magnetic field on the CE between the DI water and different solids. **a** The experimental setup and magnetization of the O_2_ molecules and ferrimagnetic samples. **b** The surface potential of the Fe_3_O_4_ sample in the DI water (O_2_ concentration, 2.5 mg L^−1^) with the magnetic field (0.5 T) switched off and on. The effect of (**c**) upward, (**d**) downward, (**e**) rightward and (**f**) leftward magnetic fields on the charge transfer between the different solid samples and the DI water (O_2_ concentration, 2.5 mg L^−1^). B denotes the magnetic field; I is the current and ∆V denotes the change of surface potential. The shaded areas around the data point indicate error bars. (Error bar are defined as s. d.) Source data are provided as a Source Data file.

Flat Fe_3_O_4_ and CoFe_2_O_4_ thin films deposited on highly doped silicon wafers were used as the solid ferrimagnetic samples, while SiO_2_ thin films were used as the non-magnetic samples for control experiments. During CE, deionized (DI) water and different organic liquids were used as the liquid contact pairs. As shown in Fig. 1a, the ferrimagnetic sample was loaded into a liquid cell with a temperature controller that regulated system temperature from 293 K to 333 K. The liquid cell was then filled with fluid, after which the charges were generated on the solid sample surface due to CE between the solid sample and the liquid. The triboelectric charges on the solid sample surface were detected directly using DH-KPFM, an open-loop KPFM mode. An alternating (AC) bias at a frequency of ω (the resonant frequency of the cantilever) was applied between the tip and the sample to drive the tip cantilever vibration. Unlike in traditional KPFM mode, the surface potential of a sample in DH-KPFM mode is calculated using the amplitude of the cantilever at the ω and 2ω frequencies and the vibrational phase shift of the cantilever at the ω frequency, instead of applying a direct (DC) compensation bias (more details about the principle of DH-KPFM is introduced elsewhere)^37,38^. Since no DC bias is applied, the ions or polar molecules in the liquid are in a quasi-static state during measurement and do not migrate or decompose. Therefore, DH-KPFM can be utilized in liquids, even polar liquids, such as DI water^39^. An electromagnetic coil was mounted below the liquid cell to generate a vertical magnetic field at the liquid–solid interface (the experimental setup for generating a horizontal magnetic field is shown in Supplementary Fig. 1). As shown in the inset in Fig. 1a, the magnetic domains of the ferrimagnetic samples were aligned with the applied magnetic field, while the three possible spin eigenstates (T_+1_, T_−1_, and T_0_) of the isolated O_2_ molecules exposed to a relatively weak magnetic field were roughly equally populated at a temperature of 293 K (Supplementary Note 1). Although the magnetic moments of O_2_ molecules are not aligned by the magnetic field, the magnetic domain alignment of the ferrimagnetic samples may change the electron transfer behavior at the interface. Furthermore, exposing the interface to magnetic fields may also affect spin-selected electron transfer.

Figure 1b illustrates the measured surface potential of the Fe_3_O_4_ sample in DI water (with a 2.5 mg L^−1^ O_2_ concentration) before and after the 0.5 T magnetic field was turned on. Before initiating the magnetic field, the Fe_3_O_4_ surface potential in DI water was about 525 mV, implying that the Fe_3_O_4_ was positively charged while in contact with the DI water (the initial Fe_3_O_4_ surface potential before being immersed in DI water was measured as about −8 mV). The positivity of the Fe_3_O_4_ surface potential increased (about 825 mV) when the magnetic field was turned on. This indicated an increase in the positive charge transference from the DI water to the F_3_O_4_ surface exposed to a magnetic field. The magnetic field-induced surface potential change was about 300 mV. Furthermore, two additional solid samples (CoFe_2_O_4_ and SiO_2_) were used in the experiments. The magnetic hysteresis loops of the three solid samples are shown in Supplementary Fig. 2. The saturated magnetic moments of the samples appeared in the following order: Fe_3_O_4_ > CoFe_2_O_4_ > SiO_2_. The CE between the three solid samples and DI water containing a 2.5 mg L^−1^ O_2_ concentration was observed in upward magnetic fields of different strengths. The magnetic field-induced surface potential changes in the samples were measured, and the magnetic field-induced transferred charge densities on the sample surfaces were calculated, as shown in Fig. 1c (the relation between the surface potential and the surface charge density was described elsewhere)^12^. A stronger magnetic field increased the transferred charge densities on the Fe_3_O_4_ and CoFe_2_O_4_ surfaces in contact with the DI water, showing higher values of up to 65 μC m^−2^ and 55 μC m^−2^, respectively, in a 0.5 T magnetic field. The effect of the magnetic field on the CE between the DI water and the SiO_2_ sample was not significant, due to the small saturated magnetic moment of the SiO_2_ sample, suggesting that the impact of the magnetic field on the liquid–solid CE is primarily related to the magnetic moment strength of the solids. The CE between the DI water and different samples were measured in droplet mode by dripping the water on a solid surface^12^. The results also indicated that a stronger magnetic field increased the charge transfer at the DI water and ferrimagnet interfaces, the same as in immersion charging mode without separation (Supplementary Fig. 3).

The effect of the magnetic field direction is also discussed here. Downward, rightward and leftward magnetic fields were applied to the liquid–solid interfaces during the experiments, as shown in Fig. 1d, e, f. The results indicated that the impact of the magnetic field on liquid–solid CE was independent of the magnetic field direction. Magnetic fields in different directions promoted the charge transfer between the DI water and the Fe_3_O_4_ and CoFe_2_O_4_ samples. This implies that the charge transfer changes are not caused by the magneto-conversion effect induced by the Lorentz force, which is direction-dependent^40^, Some other common magnetic phenomena acting at the ion-containing solution and non-ferrimagnetic sample interfaces, such as the magnetothermal effect^41^, and the Kelvin force effect^42^, are not responsible for the results since no magnetic field-induced charge transfer is observed in the control group (the CE between DI water and SiO_2_). Finally, only spin-elected electron transfer is suspected of facilitating the magnetic field effect on CE between the liquid and ferrimagnetic solids. Radical pairs with spin-correlated unpaired electrons may form at the O_2_-containing liquid and the ferrimagnet interfaces. The spin configuration conversion of the radical pairs (such as triplet-singlet spin conversion, T-S spin conversion) can be promoted by the external magnetic field via Zeeman interaction, increasing spin-selected electron transfer during liquid–solid CE^24–26^.

### Contribution of O_2_ to the magnetic sensitive charge transfer

**Fig. 2:**
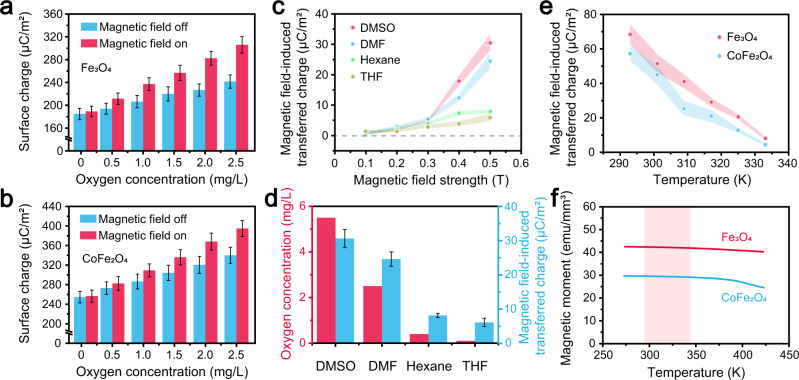
The contribution of O_2_ to the CE between liquids and ferrimagnets in a magnetic field. **a** The effect of the O_2_ concentration in the DI water on the charge transfer between the (**a**) Fe_3_O_4_ and (**b**) CoFe_2_O_4_ samples and the DI water. **c** The effect of the magnetic field on the CE between the Fe_3_O_4_ sample and different organic solutions. **d** The relationship between the O_2_ concentrations in the organic solutions and the magnetic field-induced charge transfer between the Fe_3_O_4_ sample and the organic solutions. **e** The magnetic field-induced charge transfer between the Fe_3_O_4_ and CoFe_2_O_4_ samples and the DI water at different temperatures. **f** The effect of temperature on the magnetic moments of the Fe_3_O_4_ and CoFe_2_O_4_ samples in a 0.5 T magnetic field. The shaded areas around the data point indicate error bars. (Error bar are defined as s. d.) Source data are provided as a Source Data file.

The contribution of the dissolved O_2_ molecules to the magnetic field-induced charge transfer between the liquids and ferrimagnetic solids was investigated. N_2_-saturated DI water and Ar saturated DI water with O_2_ concentrations close to 0 mg L^−1^ were used as control groups in the experiments, indicating that the surface potential of the Fe_3_O_4_ sample remained unaffected by the magnetic field, as shown in Supplementary Fig. 4. This confirmed that the dissolved N_2_ and Ar molecules did not contribute to liquid–solid CE. Figure 2a, b illustrate the triboelectric charge densities on the Fe_3_O_4_ and CoFe_2_O_4_ surfaces after contact with DI water (with different O_2_ concentrations). The positive transferred charge densities on the Fe_3_O_4_ and CoFe_2_O_4_ surfaces increased at higher O_2_ concentrations in the DI water. This implies that the dissolved O_2_ molecules are involved in the CE between the DI water and the Fe_3_O_4_ and CoFe_2_O_4_ samples. The positive transferred charge densities on the sample surfaces were consistently higher when the magnetic field was switched on than when it was off. Moreover, the charge transfer between the Fe_3_O_4_, CoFe_2_O_4_ samples and the DI water increased more rapidly in conjunction with higher O_2_ concentrations when the magnetic field was on than when it was off. This indicated that the dissolved O_2_ molecule activity increased during CE between the DI water and the ferrimagnetic samples when the magnetic field was turned on, suggesting the contribution of dissolved O_2_ molecules in the magnetic field-induced charge transfer between the liquids and the ferrimagnetic samples.

The experiments used different organic solutions, including dimethyl sulfoxide (DMSO), dimethylformamide (DMF), hexane, and tetrahydrofuran (THF) to further verify the contribution of O_2_ molecules to magnetic field-induced liquid–solid CE. Figure 2a shows the magnetic field-induced triboelectric charge density on the Fe_3_O_4_ surface in the contact with different organic liquids. The magnetic field was found to promote the Fe_3_O_4_ sample to receive positive charges in the contact with different organic liquids, as was the case with CE between Fe_3_O_4_ and DI water. The magnetic field-induced charge transfer was highest between Fe_3_O_4_ and DMSO and lowest between Fe_3_O_4_ and THF, which could be attributed to the different O_2_ affinities of the organic liquids. The O_2_ concentrations of the organic liquids are shown in Fig. 2d. The magnetic field-induced charge transfer between Fe_3_O_4_ and different organic solutions at a 0.5 T magnetic field was also provided, showing a significant correlation to the O_2_ concentrations in these liquids. The magnetic field-induced charge transfer increased at a higher O_2_ concentration, confirming that dissolved O_2_ molecules played a crucial role during CE.

Figure 2e shows the impact of temperature on the CE between the Fe_3_O_4_ and CoFe_2_O_4_ samples and the DI water containing a 2.5 mg L^−1^ O_2_ concentration at a 0.5 T magnetic field. The transferred charge density produced by the magnetic field decreased as the temperature rose from 293 K to 333 K, which did not affect the magnetic moments of Fe_3_O_4_ and CoFe_2_O_4_ when exposed to a 0.5 T magnetic field, as shown in Fig. 2f. However, the interaction between two molecules was temperature-sensitive. The electron transfer between two atoms can only occur when the electron clouds of these two atoms overlap^43^, which is achieved when two molecules collide with each other. This is considered a transient process of adsorption and desorption, with the electron transfer occurring during the former. A reasonable explanation is that the magnetic field-induced electron transfer at the O_2_-containing liquid and ferrimagnet interfaces is a relatively lengthy process. A higher temperature intensified the thermal motion of the molecules, reducing the adsorption time of two molecules at the interface, and further decreasing the probability of magnetic field-induced electron transfer between the O_2_-containing liquids and ferrimagnetic samples. As expected, these findings confirmed that the dissolved O_2_ molecules contributed to the magnetic field-induced CE between liquids and ferrimagnetic solids.

### The cycle tests of the magnetic field effect

**Fig. 3:**
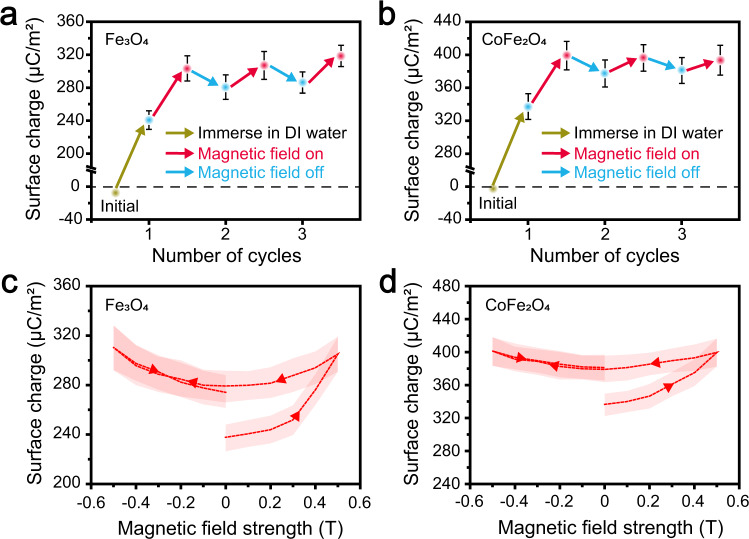
The cycle tests of the magnetic field effect on the CE between the DI water and ferrimagnetic solids. The surface charge densities of the (**a**) Fe_3_O_4_ and (**b**) CoFe_2_O_4_ samples in contact with the DI water (O_2_ concentration, 2.5 mg L^−1^) during the magnetic field (0.5 T) on and off-cycle tests. The hysteresis measurements of the magnetic field effect on the CE between the (**c**) Fe_3_O_4_ and (**d**) CoFe_2_O_4_ samples and the DI water. The shaded areas around the data point indicate error bars. (Error bar are defined as s. d.) Source data are provided as a Source Data file.

The experiments showed that the magnetic field promoted electron transfer between the O_2_-containing liquid and the ferrimagnetic sample. The spin-selective electron transfer in the radical pairs and the magnetic domain alignment of the ferrimagnetic samples are suspected to be responsible for the magnetic field-induced electron transfer. When the magnetic field was turned off, the magnetic moment of the ferrimagnetic samples decreases significantly, corresponding to a less ordered state, and the spin conversion of the radical pairs slowed down. This raises the question of whether the magnetic field-promoted electron transfer from the ferrimagnetic sample surfaces to the liquid will resume to the ferrimagnetic sample surfaces when the magnetic field is turned off. This is important for confirming the mechanism behind the magnetic field effect in CE. Figure 3a, b show the cycle tests of the magnetic field effect on the CE between the DI water (2.5 mg L^−1^ O_2_ concentration) and the Fe_3_O_4_ and CoFe_2_O_4_ samples, respectively. During these cycle tests, the initial surface charge densities of the samples were measured in air, after which it was measured again when the sample was immersed in DI water without a magnetic field. The data showed that the two solid samples received positive charges after contacting with the DI water. As expected, when the magnetic field (0.5 T) was turned on, more positive charges were transferred from the DI water to the solid surfaces. When the magnetic field was turned off, the surface charge densities of the solid samples decreased but did not reach to the value before the magnetic field was first turned on. Two more cycles were tested, showing that the electron transfer promoted by the magnetic field was mostly irreversible.

The hysteresis measurement results of the magnetic field effect on the CE between the DI water and the Fe_3_O_4_ sample are shown in Fig. 3c. The positive charge density on the Fe_3_O_4_ sample surface increased from about 240 μC m^−2^ to about 300 μC m^−2^ at a stronger magnetic field and decreased to about 280 μC m^−2^ when the magnetic field strength decreased to 0. An antiparallel magnetic field (negative magnetic field) was further applied at the Fe_3_O_4_ sample and DI water interface, increasing the surface charge density to about 300 μC m^−2^. When the antiparallel magnetic field was removed, the surface charge density returned to about 280 μC m^−2^. The hysteresis measurement of the magnetic field effect on the CE between the DI water and the CoFe_2_O_4_ sample is shown in Fig. 3d. As shown in Supplementary Fig. 2, the remanent magnetic moment of Fe_3_O_4_ during the magnetic hysteresis loop test was only about one-fourth of its saturation magnetic moment, while that of CoFe_2_O_4_ was only one-third. This indicated that the local magnetic fields on the Fe_3_O_4_ and CoFe_2_O_4_ surfaces induced by remanent magnetization were much smaller than that produced by a 0.2 T magnetic field, which can induce a saturation magnetic moment on both material surfaces. As shown in Fig. 3c, d, the electron transfer induced by the 0.2 T magnetic field increased slightly (about 5 μC m^−2^), while the irreversible electron transfer after the first magnetic field application was about 40 μC m^−2^. This suggests that irreversible electron transfer during the hysteresis measurement is unlikely due to the remanent magnetization of Fe_3_O_4_ and CoFe_2_O_4_. The contact electrification between DI water and magnetised (with 0.5 T magnetic field)/non-magnetised ferrimagnetic samples was measured to verify that the irreversible electron transfer during the hysteresis measurement is not due to the remanent magnetization. The results in Supplementary Fig. 5 show that the CE between DI water and the ferrimagnetic samples are not affected by the magnetization of the materials, which supports our analyses.

### The spin-selected electron transfer model

Based on the experimental results, DI water was chosen as representative of the O_2_-containing liquid to discuss the spin-selected electron transfer during the CE between O_2_-containing liquid and ferrimagnetic solid. During the experiments, both the Fe_3_O_4_ and CoFe_2_O_4_ ferrimagnetic samples received more positive charges in the CE with the DI water when exposed to a magnetic field, suggesting that the magnetic field facilitated electron transfer from the ferrimagnetic sample surface to the O_2_-containing DI water. In this process, the O_2_ molecules acted as acceptors for electrons from the solid sample surfaces during CE. When the O_2_ molecules in the water received the electrons, they will further take protons from the H_2_O molecules to produce OH^−^, which was similar to the process that occurred during ORR^44^. According to the Pauli exclusion principle and spin conservation principle, the spin of the transferred electrons must be antiparallel to the unpaired electrons belonging to O_2_. The entire O_2_-containing liquid–solid CE process during the experiments can be expressed as follows.

1 $$\uparrow {{{\mathrm{O}}}}={{{\mathrm{O}}}} \uparrow+\downarrow {e}^{-}(donated \, by \, F{e}_{3}{O}_{4},CoF{e}_{2}{O}_{4})+{H}_{2}O \iff \uparrow {{{\mathrm{O}}}}= {{{\mathrm{O}}}} \uparrow \downarrow {{{\mathrm{H}}}}+{{{\mathrm{O}}}}{{{\mathrm{H}}}}^{-}$$

2 $$\uparrow {{{\mathrm{O}}}}={{{\mathrm{O}}}} \uparrow \downarrow {{{\mathrm{H}}}}+\downarrow {e}^{-}(donated \, by \, F{e}_{3}{O}_{4},CoF{e}_{2}{O}_{4})+{H}_{2}O \iff {{{\mathrm{H}}}} \downarrow \uparrow {{{\mathrm{O}}}}={{{\mathrm{O}}}} \uparrow \downarrow{{{\mathrm{H}}}}+{{{\mathrm{O}}}}{{{\mathrm{H}}}}^{-}$$

During the first step (Eq. 1), the O_2_ molecules directly received electrons from the ferrimagnetic samples to produce HO_2_. Take Fe_3_O_4_ as an example, the 3d^6^ electron belonging to the Fe^2+^ in Fe_3_O_4_, which is delocalized and unpaired^45^, and the O_2_ molecule with two unpaired electrons were considered a triplet-radical pair (Supplementary Fig. 6)^46,47^. The HO_2_ contained an unpaired electron and was in a doublet state, requiring the spin configuration of the O_2_−3d^6^ electron triplet-radical pair to be converted to a doublet before electron transfer. However, the spin conversion of the O_2_−3d^6^ electron triplet-radical pair was not magnetic field sensitive due to the large zero-field splitting (ZFS) in the O_2_ molecule (the calculation details are shown in Supplementary Note 2).

During the second step (Eq. 2), the HO_2_ with one unpaired electron and a 3d^6^ electron belonging to Fe_3_O_4_ was considered a radical pair [HO_2_• •e^−^], in which the HO_2_ molecule was a free radical without ZFS. A problem is that the spin of the 3d^6^ electron in Fe_3_O_4_ was fixed by the exchange interactions, destroying the sensitivity of the radical pair to the magnetic field (Supplementary Note 3). However, a 0.5 V AC bias was applied to the liquid and solid electrodes during the KPFM measurements, resulting in the fluctuation of the 3d^6^ electrons at the interface. This fluctuation detached the 3d^6^ electrons from the solid surface, which were dissolved as water clusters. This rendered the electron transfer magnetic field sensitive, and the fluctuation should be highly associated with the liquid–solid conductivity. The DI water conductivity surpassed that of the organic solutions, the electron fluctuation induced by the AC bias at the DI water and solid interface should be stronger than that at the organic solution and solid interface. Therefore, the CE between the DI water and Fe_3_O_4_ was more sensitive to magnetic fields than that of organic solution and Fe_3_O_4_ in our experiments. At a 0.5 T magnetic field, the electron transfer at the interface consisting of the DI water with a 2.5 mg/L O_2_ concentration and Fe_3_O_4_ increased up to 65 μC m^−2^, while that comprising DMSO with a 5.5 mg/L O_2_ concentration and Fe_3_O_4_ only increased up to 30 μC m^−2^, though the former has a lower O_2_ concentration (Fig. 2). In order to further verify the importance of [HO_2_• •e^−^] radical pair in the magnetic field-induced electron transfer, the superoxide dismutase (SOD, bovine, Sigma-Aldrich)^48^, a scavenger of ${O}_{2}^{-}$, was added to the DI water to scavenge ${O}_{2}^{-}$, preventing the formation of HO_2_ and further reducing the number of [HO_2_• •e^−^] radical pair. With the increase of the SOD concentration, the number of [HO_2_• •e^−^] radical pairs decreases, and it is shown that the magnetic field sensitivity of the electron transfer between water and ferrimagnetic samples becomes weaker, which proves that the [HO_2_• •e^−^] radical pairs play an important role in magnetic field-induced electron transfer at water and ferrimagnetic solid interface (Supplementary Fig. 7).

**Fig. 4:**
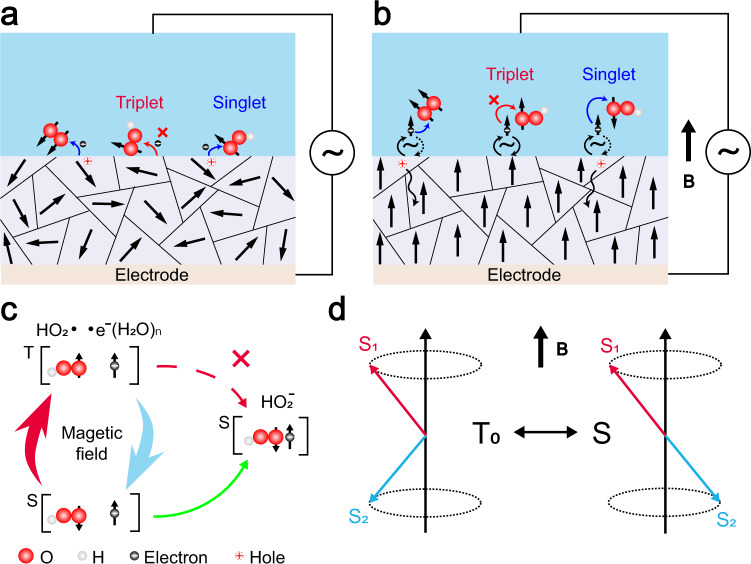
The spin-selected electron transfer model for liquid–solid CE. The spin-selected electron transfer at the O_2_-containing liquid and ferrimagnet interface (**a**) without a magnetic field and (**b**) with a magnetic field. **c** The magnetic field-induced T-S spin conversion of the [HO_2_• •e^−^] radical pair. **d** The vector representation of the T_0_-S conversion of the [HO_2_• •e^−^] radical pair. B denotes the magnetic field strength.

As shown in Fig. 4a, the magnetic domains of the ferrimagnetic samples were in a disordered state without a magnetic field, there is no magnetic field-induced spin conversion of the [HO_2_• •e^−^] pair occurs. Therefore, the electrons can be transferred from the Fe_3_O_4_ surface to the O_2_ or HO_2_ molecules only when the spin of the unpaired electrons belonging to these molecules happened to be antiparallel to the spin of the 3d^6^ electrons. As shown in Fig. 4b, the magnetic domains of the ferrimagnetic samples were aligned with the applied magnetic field, increasing its electrical conductivity. When the AC bias was applied in this case, some 3d^6^ electrons of Fe_3_O_4_ escaped from the Fe_3_O_4_ surface, becoming free dissolved electrons for half the cycle time of the AC bias. Both the HO_2_ molecule and 3d^6^ electrons were considered free radicals during this time. Moreover, the T-S spin conversion of the radical pair [HO_2_• •e^−^] was triggered by the external magnetic field at a $\triangle g{\mu }_{B}B{\hbar }^{-1}$ conversion rate ($\triangle g$ signified the difference between the $g$ factors of the HO_2_ and H_2_O clusters, ${\mu }_{B}$ denoted the Bohr magneton, $\hbar$ is the reduced Planck’s constant and $B$ represented the external magnetic field), as described in the RPM (Fig. 4c), and the calculations are shown in Supplementary Note 4^21–23^. The vector representation of the T-S conversion of the [HO_2_• •e^−^] radical pair is provided in Fig. 4d. The [HO_2_• •e^−^] pair displayed a faster spin conversion rate at a higher magnetic field and a higher electron transfer probability at the O_2_-containing DI water and ferrimagnetic sample interface, increasing the electron transfer in the presence of a magnetic field.

Both the temperature effect and cycle tests of the magnetic field effect can be explained according to the proposed spin-selected CE model. Electron transfer could only occur when the T-S spin conversion was completed within the lifetime of the [HO_2_• •e^−^] pair. The magnetic field promoted the CE between DI water and ferrimagnetic solid by accelerating the spin conversion of the [HO_2_• •e^−^] pair via the Zeeman interaction. The increasing of the temperature intensified the thermal motion of the molecules, reducing the lifetime of the [HO_2_• •e^−^] pair at the interface. Therefore, the spin conversion failed to complete even in the presence of a magnetic field, destroying the magnetic sensitivity of CE. The irreversible magnetic field-induced electron transfer during the cycle test experiments was caused by two reasons. When exposed to a magnetic field, the magnetic domains of the ferrimagnetic samples were aligned, and the holes on the sample surfaces penetrated a certain depth below the surface. When the magnetic field was removed, the less ordered state prevented the holes from returning to the solid surface due to insufficient conductivity. On the other hand, switching the magnetic field off terminated the spin evolution and prevented the back transfer of electrons. Therefore, the effect of the magnetic field on liquid–solid CE is irreversible.

The magnetic field-controlled CE between the O_2_-containing liquid and the Fe_3_O_4_ and CoFe_2_O_4_ samples demonstrated the presence of electron transfer during liquid–solid CE, supporting the “two-step” model for the formation of the hybrid EDL^49^. Moreover, it is an interesting observation that the surface of ferrimagnetic samples donated electrons in the experiments, since that the surface of most solids is tend to be negatively charged in contact with water. This supports the spin electron transfer at liquid–solid interface. The electrons in ferrimagnetic materials spontaneously polarize due to exchange interaction, forming magnetic domains at the microscale. In these microdomains, the electron spins are ordered, while the electron spins in water are disordered. The transfer of electrons from the water side to the ferrimagnetic solid side represents a process from a spin disorder state to a spin order state, which is difficult to occur from a thermodynamic perspective. However, the electron transfer from the ferrimagnetic solid side to the water side represents a process from a spin order state to a spin disorder state, which is consistent with the principle of entropy increase. Consequently, water tends to gain electrons when in contact with a ferrimagnetic solid, while the ferrimagnetic solid tends to lose electrons and receive positive charges. The results also indicated that liquid–solid CE was fundamentally a spin-selected chemical reaction, in which electron transfer occurred as the first step. Unlike traditional chemical reactions, the transferred electrons or holes usually accumulate on the solid surface, reaching saturation during liquid–solid CE, consequently preventing further electron transfer, such as a chemical reaction with “negative feedback”. This accumulation attracted opposite ions in the liquid to form an EDL. Since the electron transfer during liquid–solid CE was spin-selected, the density of the accumulated electrons or holes on the solid surface was regulated by the magnetic field, further controlling the EDL structure at the liquid–solid interface. This provides an approach for controlling chemical reactions in EDL-related areas, such as mechanochemistry, electrocatalysis, electrochemical storage, and electrophoresis.

## Discussion

In conclusion, the CE between the Fe_3_O_4_ and CoFe_2_O_4_ samples and different O_2_-containing liquids is observed when exposed to a magnetic field, while the contribution of the dissolved O_2_ molecules in the solution to the liquid–solid CE is investigated. Magnetic fields promote positive charge transfer from the O_2_-containing liquid to the Fe_3_O_4_ and CoFe_2_O_4_ sample surfaces, increasing in conjunction with higher O_2_ concentrations in the liquid and decreasing with elevated temperatures. These results suggest that the spin configuration of the [HO_2_• •e^−^] pairs is affected by the magnetic field, promoting electron transfer during liquid–solid CE. This implies the presence of spin-selected electron transfer during liquid–solid CE. A spin-selected electron transfer model is proposed based on the RPM, in which the HO_2_ molecules and the unpaired electrons belonging to the ferrimagnetic solids are considered radical pairs, while the magnetic field can accelerate the triplet-singlet spin conversion of the [HO_2_• •e^−^] pairs. These findings provide strong evidence for electron transfer during CE at the liquid–solid interface, presenting significant implications for EDL-related fields.

## Methods

### Sample preparation and characterization

Fe_3_O_4_ and CoFe_2_O_4_ layers of 100 nm thick were deposited via magnetron sputtering on silicon wafers highly doped with boron. The SiO_2_ layer (100 nm thick) was prepared via thermal oxidation on boron-doped silicon wafers. Since the SiO_2_ sample consisted of non-magnetic material, its small saturated magnetic moment might be caused by the doping elements. DI water with a resistivity of 18.2 MΩ ∙ cm was obtained using a deionizer (HHitech, China). The DI water with specific O_2_ concentrations was prepared by mixing O_2_ saturated DI water with cold, boiled DI water, with an O_2_ concentration close to 0. The ferrimagnetic samples before and after contact with DI water under 0.5 T magnetic field by using X-ray photoelectron spectroscopy (XPS), the results are shown in Supplementary Figs. 8 and 9. The results show that there is no change in the ferrimagnetic sample surface before and after contact electrification, suggesting that the charge transfer between DI water and the ferrimagnetic sample, and irreversible charge transfer in the cycle tests of the magnetic field effect are not caused by the oxidization of the solid surfaces.

### DH-KPFM experiments

The KPFM experiments were performed using a Dimension Icon commercial AFM/KPFM system (Bruker, USA) and a conductive SCM-PIT tip (Bruker, USA; coating: Pt/Ir; resonance frequency: 75 kHz; spring constant: 3 N/m). Here, the conductive tip should not be magnetic, since they will be subjected to a significant magnetic force, yielding a significant cantilever deflection under magnetic field, further affecting the potential signal, as shown in Supplementary Fig. 10 and Supplementary Note 5. Before the DH-KPFM experiment, the Q factor of the tip was measured in liquid at the ω (the resonant frequency of the cantilever) and 2ω frequencies. During the DH-KPFM experiment, the topography of the sample was first measured in PeakForce tapping mode, the peakforce was set to 300 pN, to make sure that there is no charge transfer introduced in PeakForce tapping scanning (Supplementary Fig. 11 and Supplementary Note 6). The tip was then lifted 50 nm for a second topographical scan, during which a 500 mV AC bias was applied to the tip and sample to drive the cantilever vibration. The cantilever amplitude at the ω and 2ω frequencies and the cantilever phase shift at the ω frequency were recorded. The surface potential was ultimately calculated as following^37^:

3 $$V=\frac{{A}_{\omega }{\cos }({\theta }_{\omega })}{{A}_{2\omega }}\frac{{V}_{{ac}}}{4{X}_{{gain}}}$$

Where *V* is the surface potential, ${A}_{\omega }$ and ${A}_{2\omega }$ denote the amplitude of the tip at the ω and 2ω frequencies, ${\theta }_{\omega }$ is the phase shift at the ω frequency, ${V}_{{ac}}$ is the amplitude of the applied AC bias, and ${X}_{{gain}}$ denotes the ratio between two Q factor of the tip at the ω (the resonant frequency of the cantilever) and 2ω frequencies.

The calculations show that the Ampere’s force experienced by the Pt-coated AFM tip was too small to affect the potential measurement under the magnetic field, as shown in Supplementary Fig. 12 and Supplementary Note 7^50^. All experiments were performed at 293 K unless otherwise specified.

## Supplementary information

Supplementary Information

## Data Availability

All data needed to evaluate the conclusions in the paper are present in the paper and/or the Supplementary Information. Source data are provided as a Source Data file. The source data underlying all figures can be found in the Source Data file. Source data are provided with this paper.

## Source data

Source Data
