# Supplementary material for: Spin-selected electron transfer in liquid–solid contact electrification
Source: Nat Commun. 2022 Sep 5;13:5230. doi: 10.1038/s41467-022-32984-9 (PMC9445095; doi:10.1038/s41467-022-32984-9)
Supplement: Supplementary file 1 — Supplementary Information [file 41467_2022_32984_MOESM1_ESM.pdf]

Supplementary Information

Spin-selected electron transfer in liquid-solid contact electrification

Lin at al.

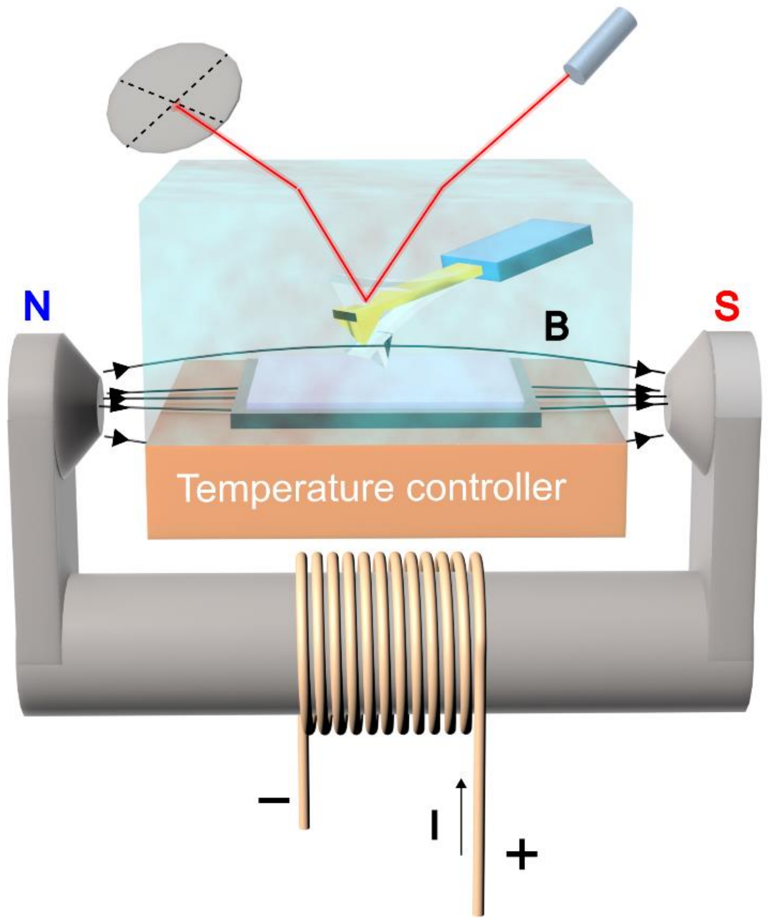

Supplementary Figure 1. The experimental setup for generating a horizontal magnetic field.

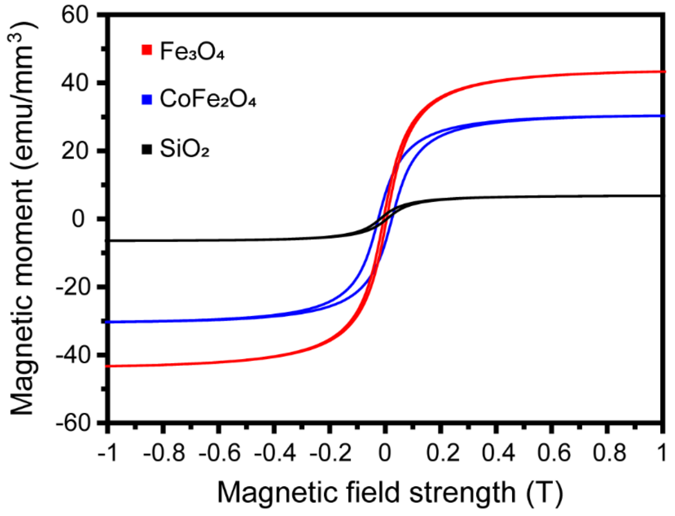

Supplementary Figure 2. The magnetic hysteresis loop tests of the different solid samples. Source data are provided as a Source Data file.

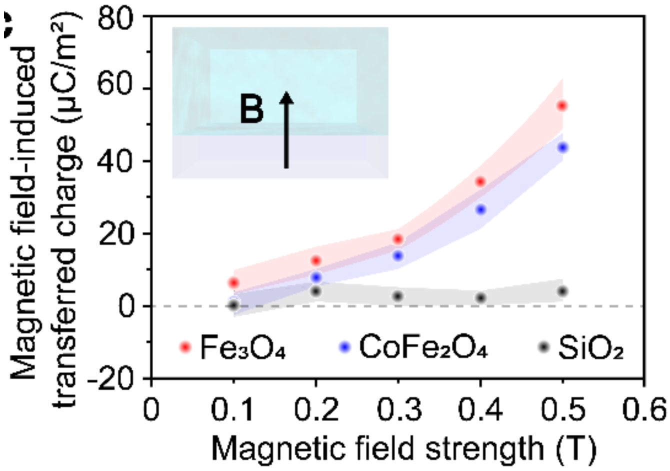

Supplementary Figure 3. The effect of a magnetic field on the charge transfer between the solid samples and the DI water (O_2 concentration, 2.5 mg/L) in droplet mode. The shaded areas around the data point indicate error bars. Error bar are defined as s. d. Source data are provided as a Source Data file.

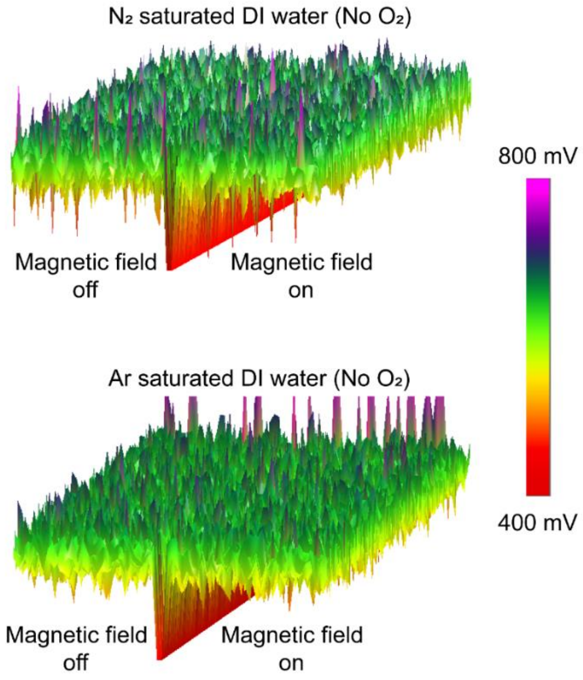

Supplementary Figure 4. The effect of a 0.5 T magnetic field on the surface potential of Fe_3O_4 in N_2 and Ar saturated DI water. Source data are provided as a Source Data file.

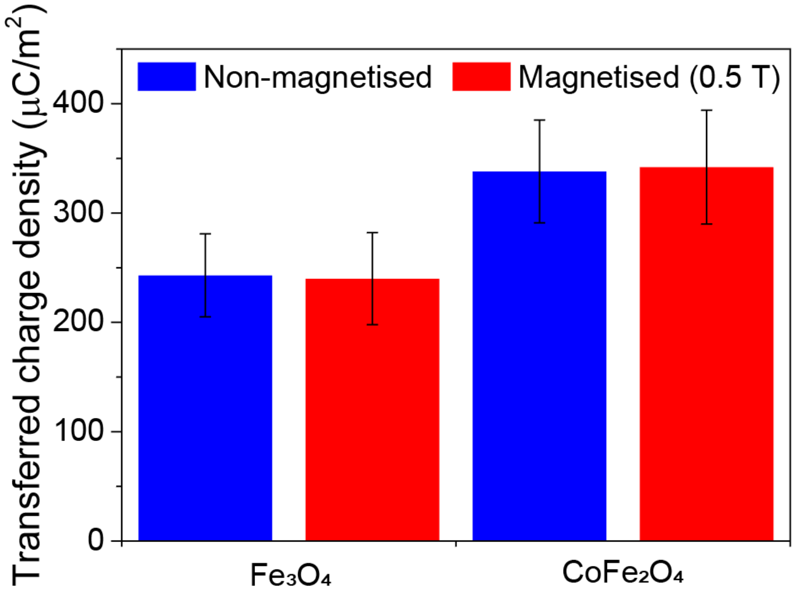

Supplementary Figure 5. The contact electrification between DI water (O_2 concentration, 2.5 mg/L) and magnetised /non-magnetised ferrimagnetic samples in droplet mode. Error bar are defined as s. d. Source data are provided as a Source Data file.

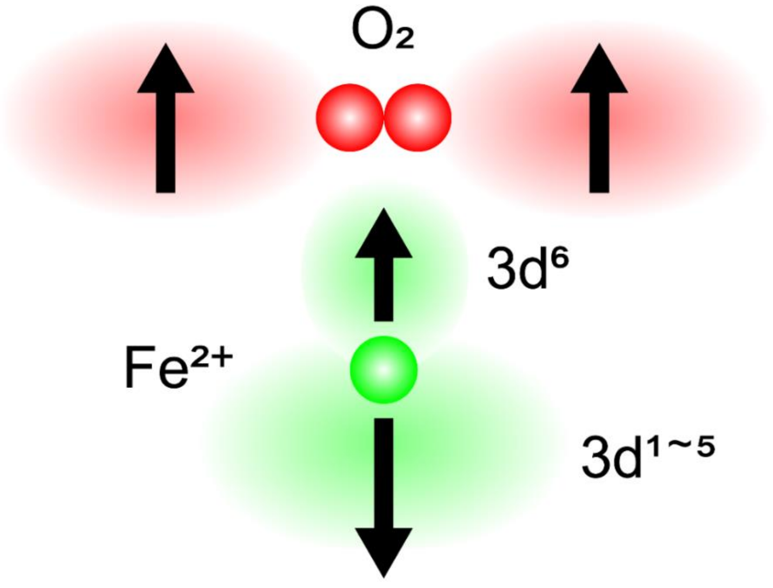

Supplementary Figure 6. A schematic of the interaction between the O_2 molecules and Fe^{2+}.

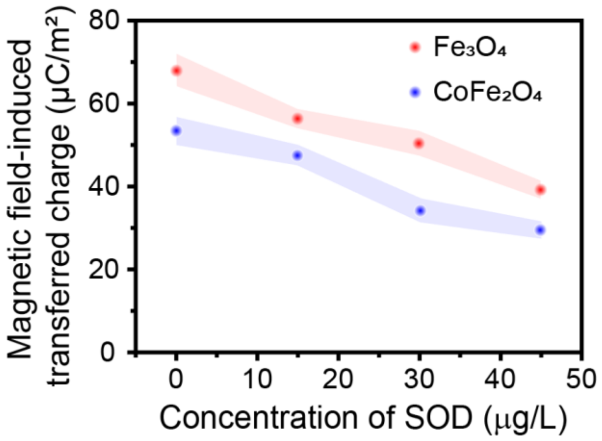

Supplementary Figure 7. The effect of SOD on the magnetic field-induced charge transfer between DI water and ferrimagnetic solids under 0.5 T magnetic field. The shaded areas around the data point indicate error bars. Error bar are defined as s. d. Source data are provided as a Source Data file.

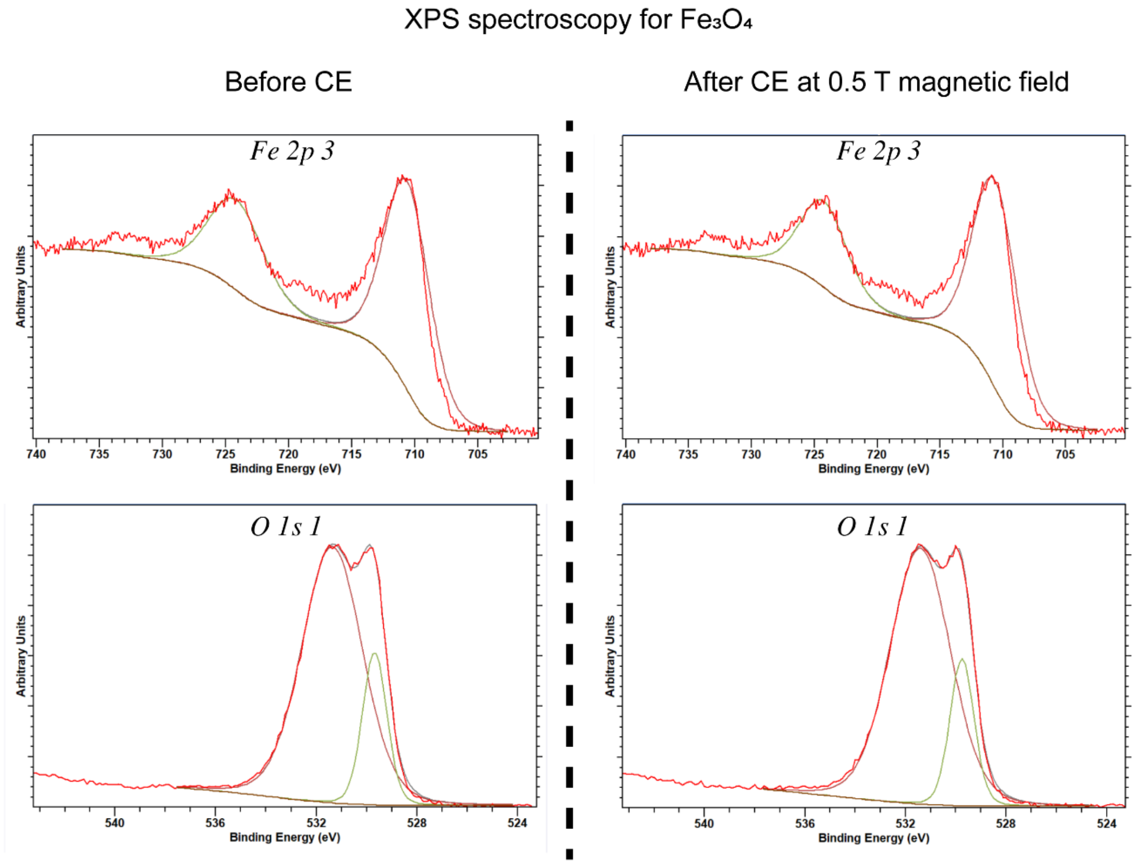

Supplementary Figure 8. The XPS spectroscopy of Fe_3O_4 samples before and after contact with DI water (O_2 concentration, 2.5 mg/L) under 0.5 T magnetic field. Source data are provided as a Source Data file.

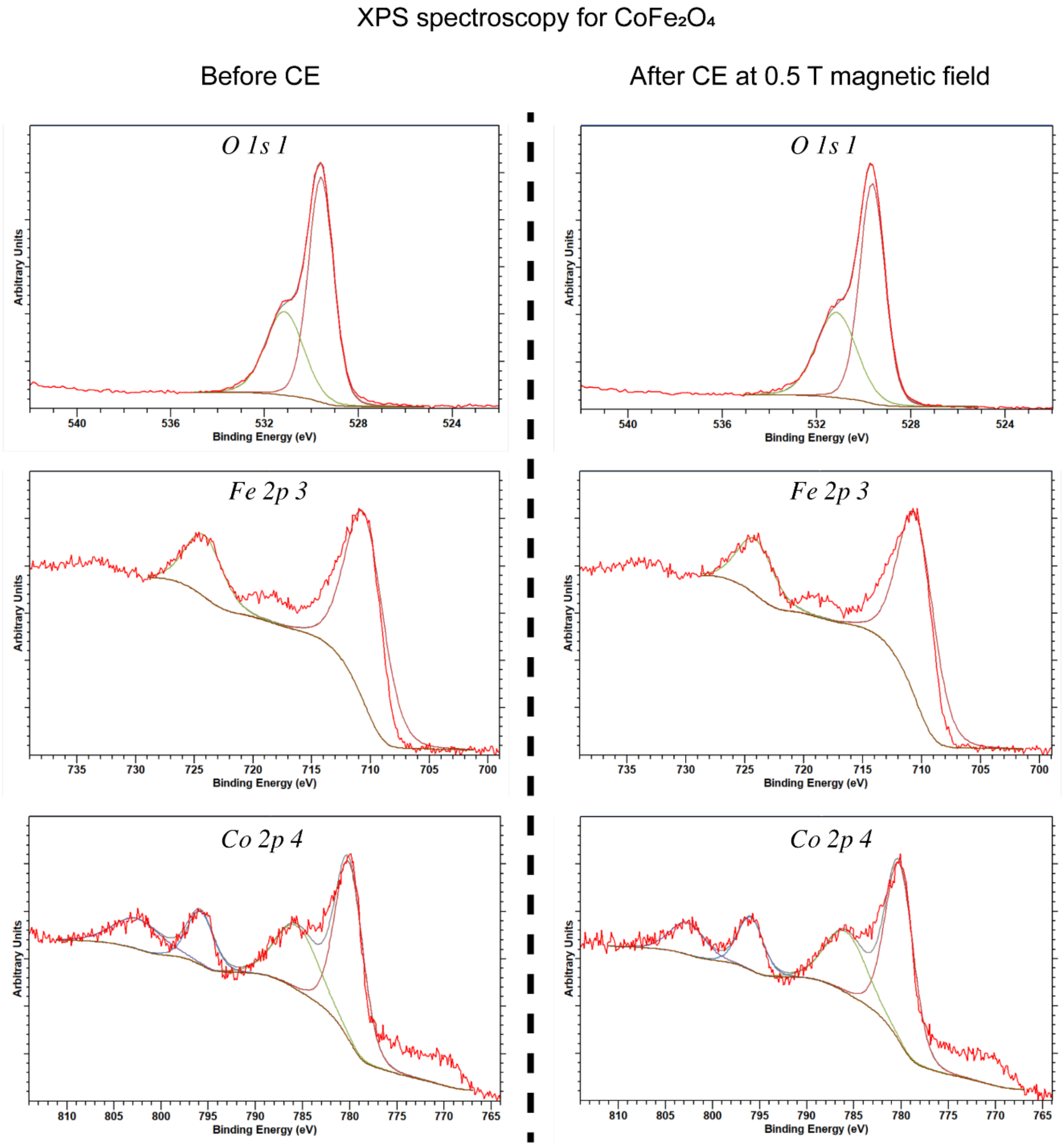

Supplementary Figure 9. The XPS spectroscopy of $CoFe_2O_4$ samples before and after contact with DI water (O_2 concentration, 2.5 mg/L) under 0.5 T magnetic field. Source data are provided as a Source Data file.

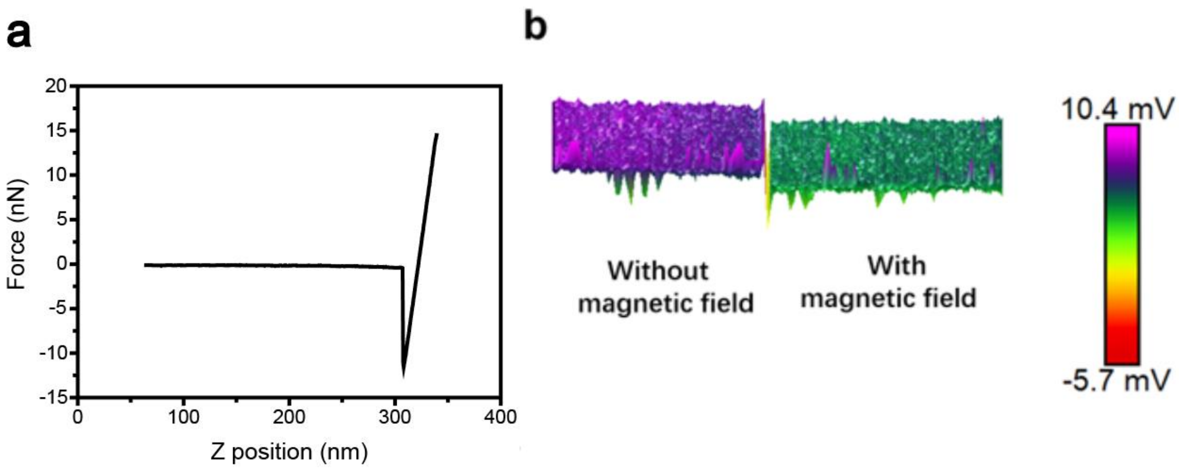

Supplementary Figure 10. (a) The force-distance curve between the magnetic tip and the Fe_3O_4 sample. (b) The "surface potential" measured using a magnetic tip. Source data are provided as a Source Data file.

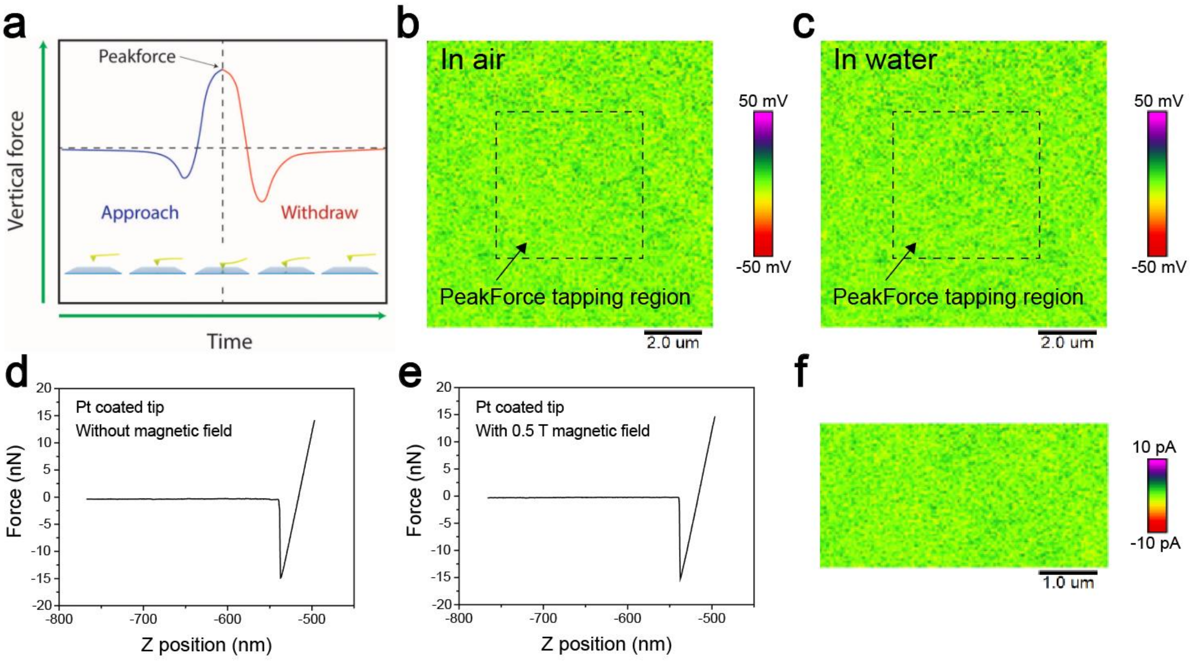

Supplementary Figure 11. (a) The force curve in PeakForce tapping mode. The charge transfer induced by PeakForce tapping mode in (b) air and (c) DI water. The force-distance curve of the Pt coated tip on the Fe_3O_4 surface (d) without magnetic field and (e) with 0.5 T magnetic field. (f) The tribo-current between the Pt coated tip and Fe_3O_4 sample in PeakForce tapping mode. Source data are provided as a Source Data file.

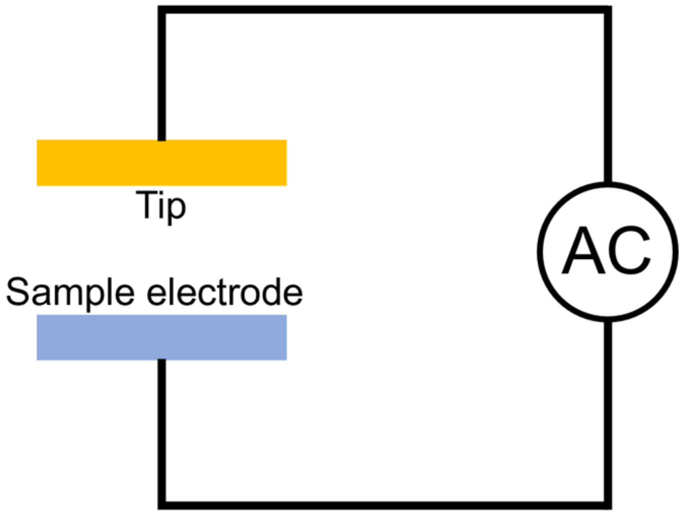

Supplementary Figure 12. The capacitance model for the DH-KPFM system.

Supplementary Note 1

The spin polarization of an O_2 molecule in a magnetic field can be defined as (ignoring the ca. 4 T zero-field splitting):

$$p = \frac{n_{\uparrow\uparrow} - n_{\downarrow\downarrow}}{n_{\uparrow\uparrow} + n_{\downarrow\downarrow}} \quad \text{(S1)}$$

where $n_{\uparrow\uparrow}$ and $n_{\downarrow\downarrow}$ are the equilibrium populations of the $m = +1$ and $m = -1$ triplet sub-levels. Using the Boltzmann distribution and g = 2 (g-value), B = 0.5 T (magnetic field strength), and T = 293 K (temperature):

$$p = \frac{e^{(g\mu_B B/k_B T)} - e^{(-g\mu_B B/k_B T)}}{e^{(g\mu_B B/k_B T)} + e^{(-g\mu_B B/k_B T)}} = 0.0023 \quad \text{(S2)}$$

where μ_B is the Bohr magneton, B is the external magnetic field and k_B is the Boltzmann's constant. The population of the $n_{\uparrow\uparrow}$ state exceeds that of the $n_{\uparrow\uparrow}$ state by 0.46% at 293 K, which can be disregarded.

Supplementary Note 2

As shown in Fig. S6, when the O_2 molecule interacts with the Fe^{2+} ion on the Fe_3O_4 surface, the $3d^6$ electrons belonging to the Fe^{2+} ion transfer to the O_2 molecule. During this process, the O_2 molecule and the $3d^6$ electron can be considered a triplet-radical pair, and the spin Hamiltonian can be expressed as follows:

$$\mathbf{H} = \mathbf{H}_{\mathbf{ZFS-O_2}} + \mathbf{H}_{\mathbf{ex}} + \mathbf{H}_{\mathbf{mag}} \tag{S3}$$

where $\mathbf{H}_{\mathbf{ZFS-O_2}}$ denotes the zero-field splitting in the O_2 molecule, $\mathbf{H}_{\mathbf{ex}}$ signifies the exchange interaction between the O_2 molecule and Fe^{2+}, and $\mathbf{H}_{\mathbf{mag}}$ represents the Zeeman interaction, and,

$$\mathbf{H}_{\mathbf{ZFS-O_2}} = D(S_{z-O_2}^2 - \frac{{S_{O_2}}^2}{3}) \tag{S4}$$

where D is a constant.

$$\mathbf{H}_{\mathbf{ex}} = -\frac{1}{4}J(r)(1 + 3\mathbf{S}_{\mathbf{T}}\mathbf{S}_{\mathbf{r}}) \tag{S5}$$

where $J(r)$ is the exchange constant and r denotes the distance between the O_2 molecule and Fe^{2+} ion.

$$\mathbf{H}_{\mathbf{mag}} = g\mu_B B S_z \tag{S6}$$

Here, the hyperfine interaction is disregarded since it is significantly smaller than the other terms. The $3d^6$ electron is considered a free radical, since the AC bias in the KPFM measurements unbinds the $3d^6$ electrons from the solid surface, becoming dissolved electrons belonging to water clusters.

The three-electron system displays four quartet configurations and four doublet configurations, as follows:

$$|Q_{3/2}\rangle = \alpha\alpha\alpha \tag{S7-1}$$

$$|Q_{1/2}\rangle = 3^{-1/2}(\alpha\alpha\beta + \alpha\beta\alpha + \beta\alpha\alpha) \tag{S7-2}$$

$$|Q_{-1/2}\rangle = 3^{-1/2}(\beta\beta\alpha + \beta\alpha\beta + \alpha\beta\beta) \tag{S7-3}$$

$$|Q_{-3/2}\rangle = \beta\beta\beta \tag{S7-4}$$

$$|D_{1/2}\rangle = 6^{-1/2}(\alpha\alpha\beta + \alpha\beta\alpha - 2\beta\alpha\alpha) \tag{S7-5}$$

$$|D_{-1/2}\rangle = 6^{-1/2}(\beta\beta\alpha + \beta\alpha\beta - 2\alpha\beta\beta) \tag{S7-6}$$

$$|D'_{1/2}\rangle = 2^{-1/2}(\alpha\alpha\beta - \alpha\beta\alpha) \tag{S7-7}$$

$$|D'_{-1/2}\rangle = 2^{-1/2}(\beta\beta\alpha - \beta\alpha\beta) \tag{S7-8}$$

The spin conversion of the two quantum states depends on the difference between the energy levels of these two states and the corresponding off-diagonal matrix elements.

Here, it is considered that $J(r) = 0$. Therefore, the energy level of different spin states can be calculated as follows (g_{O_2} and $g_{Fe_3O_4}$ are the g factors of the O_2 molecule and Fe_3O_4, respectively.):

$$E_{Q_{\frac{3}{2}}} = \left\langle Q_{\frac{3}{2}} \middle| \mathbf{H_{ZFS-O_2}} + \mathbf{H_{mag}} \middle| Q_{\frac{3}{2}} \right\rangle = \left\langle \alpha\alpha\alpha \middle| D(S_{z-O_2}^2 - \frac{{S_{O_2}}^2}{3}) + g\mu_B B(S_{z1}+S_{z2}+S_{z3}) \middle| \alpha\alpha\alpha \right\rangle = \frac{1}{3}D + \frac{1}{2}(2g_{O_2} + g_{Fe_3O_4})\mu_B B \quad \text{(S8-1)}$$

$$E_{Q_{\frac{1}{2}}} = \left\langle Q_{\frac{1}{2}} \middle| \mathbf{H_{ZFS-O_2}} + \mathbf{H_{mag}} \middle| Q_{\frac{1}{2}} \right\rangle =$$
$$\left\langle 3^{-1/2}(\alpha\alpha\beta + \alpha\beta\alpha + \beta\alpha\alpha) \middle| D(S_{z-O_2}^2 - \frac{{S_{O_2}}^2}{3}) + g\mu_B B(S_{z1}+S_{z2}+S_{z3}) \middle| 3^{-1/2}(\alpha\alpha\beta + \alpha\beta\alpha + \beta\alpha\alpha) \right\rangle =$$
$$\frac{1}{3}\left\langle \alpha\alpha\beta + \alpha\beta\alpha + \beta\alpha\alpha \middle| D(S_{z-O_2}^2 - \frac{{S_{O_2}}^2}{3}) + g\mu_B B(S_{z1}+S_{z2}+S_{z3}) \middle| \alpha\alpha\beta + \alpha\beta\alpha + \beta\alpha\alpha \right\rangle = -\frac{1}{3}D + \frac{1}{6}(2g_{O_2} + g_{Fe_3O_4})\mu_B B \quad \text{(S8-2)}$$

$$E_{Q_{-\frac{1}{2}}} = \left\langle Q_{-\frac{1}{2}} \middle| \mathbf{H_{ZFS-O_2}} + \mathbf{H_{mag}} \middle| Q_{-\frac{1}{2}} \right\rangle =$$
$$\left\langle 3^{-1/2}(\beta\beta\alpha + \beta\alpha\beta + \alpha\beta\beta) \middle| D(S_{z-O_2}^2 - \frac{{S_{O_2}}^2}{3}) + g\mu_B B(S_{z1}+S_{z2}+S_{z3}) \middle| 3^{-1/2}(\beta\beta\alpha + \beta\alpha\beta + \alpha\beta\beta) \right\rangle =$$
$$\frac{1}{3}\left\langle \beta\beta\alpha + \beta\alpha\beta + \alpha\beta\beta \middle| D(S_{z-O_2}^2 - \frac{{S_{O_2}}^2}{3}) + g\mu_B B(S_{z1}+S_{z2}+S_{z3}) \middle| \beta\beta\alpha + \beta\alpha\beta + \alpha\beta\beta \right\rangle = -\frac{1}{3}D - \frac{1}{6}(2g_{O_2} + g_{Fe_3O_4})\mu_B B \quad \text{(S8-3)}$$

$$E_{Q_{-\frac{3}{2}}} = \left\langle Q_{-\frac{3}{2}} \middle| \mathbf{H_{ZFS-O_2}} + \mathbf{H_{mag}} \middle| Q_{-\frac{3}{2}} \right\rangle = \left\langle \beta\beta\beta \middle| D(S_{z-O_2}^2 - \frac{{S_{O_2}}^2}{3}) + g\mu_B B(S_{z1}+S_{z2}+S_{z3}) \middle| \beta\beta\beta \right\rangle = \frac{1}{3}D - \frac{1}{2}(2g_{O_2} + g_{Fe_3O_4})\mu_B B \quad \text{(S8-4)}$$

$$E_{D_{\frac{1}{2}}} = \left\langle D_{\frac{1}{2}} \middle| \mathbf{H_{ZFS-O_2}} + \mathbf{H_{mag}} \middle| D_{\frac{1}{2}} \right\rangle =$$
$$\left\langle 6^{-1/2}(\alpha\alpha\beta + \alpha\beta\alpha - 2\beta\alpha\alpha) \middle| D(S_{z-O_2}^2 - \frac{{S_{O_2}}^2}{3}) + g\mu_B B(S_{z1}+S_{z2}+S_{z3}) \middle| 6^{-1/2}(\alpha\alpha\beta + \alpha\beta\alpha - 2\beta\alpha\alpha) \right\rangle =$$
$$\frac{1}{6}\left\langle \alpha\alpha\beta + \alpha\beta\alpha - 2\beta\alpha\alpha \middle| D(S_{z-O_2}^2 - \frac{{S_{O_2}}^2}{3}) + g\mu_B B(S_{z1}+S_{z2}+S_{z3}) \middle| \alpha\alpha\beta + \alpha\beta\alpha - 2\beta\alpha\alpha \right\rangle = \frac{1}{12}(g_{O_2} + 2g_{Fe_3O_4})\mu_B B \quad \text{(S8-5)}$$

$$E_{D_{-\frac{1}{2}}} = \left\langle D_{-\frac{1}{2}} \middle| \mathbf{H_{ZFS-O_2}} + \mathbf{H_{mag}} \middle| D_{-\frac{1}{2}} \right\rangle =$$
$$\left\langle 6^{-1/2}(\beta\beta\alpha + \beta\alpha\beta - 2\alpha\beta\beta) \middle| D(S_{z-O_2}^2 - \frac{{S_{O_2}}^2}{3}) + g\mu_B B(S_{z1}+S_{z2}+S_{z3}) \middle| 6^{-1/2}(\beta\beta\alpha + \beta\alpha\beta - 2\alpha\beta\beta) \right\rangle =$$
$$\frac{1}{6}\left\langle \beta\beta\alpha + \beta\alpha\beta - 2\alpha\beta\beta \middle| D(S_{z-O_2}^2 - \frac{{S_{O_2}}^2}{3}) + g\mu_B B(S_{z1}+S_{z2}+S_{z3}) \middle| \beta\beta\alpha + \beta\alpha\beta - 2\alpha\beta\beta \right\rangle = -\frac{1}{12}(g_{O_2} + 2g_{Fe_3O_4})\mu_B B \quad \text{(S8-6)}$$

$$E_{D'_{\frac{1}{2}}} = \left\langle D'_{\frac{1}{2}} \middle| \mathbf{H_{ZFS-O_2}} + \mathbf{H_{mag}} \middle| D'_{\frac{1}{2}} \right\rangle =$$

$$\left\langle 2^{-1/2}(\alpha\alpha\beta - \alpha\beta\alpha) \middle| D(S^2_{z-O_2} - \frac{{S_{O_2}}^2}{3}) + g\mu_B B(S_{z1}+S_{z2}+S_{z3}) \middle| 2^{-1/2}(\alpha\alpha\beta - \alpha\beta\alpha) \right\rangle =$$

$$\frac{1}{2}\left\langle \alpha\alpha\beta - \alpha\beta\alpha \middle| D(S^2_{z-O_2} - \frac{{S_{O_2}}^2}{3}) + g\mu_B B(S_{z1}+S_{z2}+S_{z3}) \middle| \alpha\alpha\beta - \alpha\beta\alpha \right\rangle = \frac{1}{2} g_{O_2}\mu_B B \quad \text{(S8-7)}$$

$$E_{D'_{-\frac{1}{2}}} = \left\langle D'_{-\frac{1}{2}} \middle| \mathbf{H_{ZFS-O_2}} + \mathbf{H_{mag}} \middle| D'_{-\frac{1}{2}} \right\rangle =$$

$$\left\langle 2^{-1/2}(\beta\beta\alpha - \beta\alpha\beta) \middle| D(S^2_{z-O_2} - \frac{{S_{O_2}}^2}{3}) + g\mu_B B(S_{z1}+S_{z2}+S_{z3}) \middle| 2^{-1/2}(\beta\beta\alpha - \beta\alpha\beta) \right\rangle =$$

$$\frac{1}{2}\left\langle \beta\beta\alpha - \beta\alpha\beta \middle| D(S^2_{z-O_2} - \frac{{S_{O_2}}^2}{3}) + g\mu_B B(S_{z1}+S_{z2}+S_{z3}) \middle| \beta\beta\alpha - \beta\alpha\beta \right\rangle = -\frac{1}{2} g_{O_2}\mu_B B \quad \text{(S8-8)}$$

The energy levels of all four quartet states contain the $\pm\frac{1}{3}D$ term. For the O_2 molecule, the D parameter value is about 3.5 cm^{-1}, which is significantly larger than the Zeeman term ($g\mu_B B$, where $g \approx 2,\ \mu_B = 0.927 \times 10^{-23} Am^2$ and $B = 0.5\ T$). The energy differences between the quartet and doublet states are not sensitive to the external magnetic field.

The corresponding off-diagonal matrix elements can be expressed as follows:

$$\left\langle D_{\frac{1}{2}} \middle| \mathbf{H_{ZFS-O_2}} + \mathbf{H_{mag}} \middle| Q_{\frac{3}{2}} \right\rangle = \left\langle D_{-\frac{1}{2}} \middle| \mathbf{H_{ZFS-O_2}} + \mathbf{H_{mag}} \middle| Q_{\frac{3}{2}} \right\rangle = \left\langle D'_{\frac{1}{2}} \middle| \mathbf{H_{ZFS-O_2}} + \mathbf{H_{mag}} \middle| Q_{\frac{3}{2}} \right\rangle =$$

$$\left\langle D'_{-\frac{1}{2}} \middle| \mathbf{H_{ZFS-O_2}} + \mathbf{H_{mag}} \middle| Q_{\frac{3}{2}} \right\rangle = 0 \quad \text{(S9-1)}$$

$$\left\langle D_{\frac{1}{2}} \middle| \mathbf{H_{ZFS-O_2}} + \mathbf{H_{mag}} \middle| Q_{-\frac{3}{2}} \right\rangle = \left\langle D_{-\frac{1}{2}} \middle| \mathbf{H_{ZFS-O_2}} + \mathbf{H_{mag}} \middle| Q_{-\frac{3}{2}} \right\rangle = \left\langle D'_{\frac{1}{2}} \middle| \mathbf{H_{ZFS-O_2}} + \mathbf{H_{mag}} \middle| Q_{-\frac{3}{2}} \right\rangle =$$

$$\left\langle D'_{-\frac{1}{2}} \middle| \mathbf{H_{ZFS-O_2}} + \mathbf{H_{mag}} \middle| Q_{-\frac{3}{2}} \right\rangle = 0 \quad \text{(S9-2)}$$

$$\left\langle D_{\frac{1}{2}} \middle| \mathbf{H_{ZFS-O_2}} + \mathbf{H_{mag}} \middle| Q_{\frac{1}{2}} \right\rangle =$$

$$\langle 6^{-1/2}(\alpha\alpha\beta + \alpha\beta\alpha - 2\beta\alpha\alpha) | \mathbf{H_{ZFS-O_2}} + \mathbf{H_{mag}} | 3^{-1/2}(\alpha\alpha\beta + \alpha\beta\alpha + \beta\alpha\alpha) \rangle =$$

$$\frac{\sqrt{2}}{6}\left\langle \alpha\alpha\beta + \alpha\beta\alpha - 2\beta\alpha\alpha \middle| D(S^2_{z-O_2} - \frac{{S_{O_2}}^2}{3}) + g\mu_B B(S_{z1}+S_{z2}+S_{z3}) \middle| \alpha\alpha\beta + \alpha\beta\alpha + \beta\alpha\alpha \right\rangle = \frac{\sqrt{2}}{6}(D + (g_{O_2} - g_{Fe_3O_4})\mu_B B) \quad \text{(S9-3)}$$

$$\left\langle D'_{\frac{1}{2}} \middle| \mathbf{H_{ZFS-O_2}} + \mathbf{H_{mag}} \middle| Q_{\frac{1}{2}} \right\rangle = \langle 2^{-1/2}(\alpha\alpha\beta - \alpha\beta\alpha) | \mathbf{H_{ZFS-O_2}} + \mathbf{H_{mag}} | 3^{-1/2}(\alpha\alpha\beta + \alpha\beta\alpha + \beta\alpha\alpha) \rangle =$$

$$\frac{\sqrt{6}}{6}\left\langle \alpha\alpha\beta - \alpha\beta\alpha \middle| D(S^2_{z-O_2} - \frac{{S_{O_2}}^2}{3}) + g\mu_B B(S_{z1}+S_{z2}+S_{z3}) \middle| \alpha\alpha\beta + \alpha\beta\alpha + \beta\alpha\alpha \right\rangle = \frac{\sqrt{6}}{6}(D + (g_{O_2} - g_{Fe_3O_4})\mu_B B) \quad \text{(S9-4)}$$

$$\left\langle D_{-\frac{1}{2}}\middle|\mathbf{H_{ZFS-O_2}}+\mathbf{H_{mag}}\middle|Q_{-\frac{1}{2}}\right\rangle =$$

$$\left\langle 6^{-\frac{1}{2}}(\beta\beta\alpha+\beta\alpha\beta-2\alpha\beta\beta)\middle|\mathbf{H_{ZFS-O_2}}+\mathbf{H_{mag}}\middle|3^{-1/2}(\beta\beta\alpha+\beta\alpha\beta+\alpha\beta\beta)\right\rangle =$$

$$\frac{\sqrt{2}}{6}\left\langle\beta\beta\alpha+\beta\alpha\beta-2\alpha\beta\beta\middle|D(S_{z-O_2}^2-\frac{{S_{O_2}}^2}{3})+g\mu_B B(S_{z1}+S_{z2}+S_{z3})\middle|\beta\beta\alpha+\beta\alpha\beta+\alpha\beta\beta\right\rangle=\frac{\sqrt{2}}{6}(D-(g_{O_2}-g_{Fe_3O_4})\mu_B B) \quad \text{(S9-5)}$$

$$\left\langle D'_{-1/2}\middle|\mathbf{H_{ZFS-O_2}}+\mathbf{H_{mag}}\middle|Q_{-\frac{1}{2}}\right\rangle =$$

$$\langle 2^{-1/2}(\beta\beta\alpha-\beta\alpha\beta)|\mathbf{H_{ZFS-O_2}}+\mathbf{H_{mag}}|3^{-1/2}(\beta\beta\alpha+\beta\alpha\beta+\alpha\beta\beta)\rangle =$$

$$\frac{\sqrt{6}}{6}\left\langle\beta\beta\alpha-\beta\alpha\beta\middle|D(S_{z-O_2}^2-\frac{{S_{O_2}}^2}{3})+g\mu_B B(S_{z1}+S_{z2}+S_{z3})\middle|\beta\beta\alpha+\beta\alpha\beta+\alpha\beta\beta\right\rangle=\frac{\sqrt{6}}{6}(D-(g_{O_2}-g_{Fe_3O_4})\mu_B B) \quad \text{(S9-6)}$$

It can be seen that all the off-diagonal matrix elements are either equal to zero or contain the D parameter (about 3.5 cm^{-1}). The Zeeman term is equal to $\Delta g\mu_B B$, which is several orders of magnitude smaller than the D parameter. The calculations show that both the energy differences between the quartet and doublet states and the corresponding off-diagonal matrix elements are not sensitive to magnetic fields. This suggests that the insufficient magnetic field sensitivity of the quartet-doublet spin conversion of the O_2 molecule-$3d^6$ electron triplet-radical pair.

Supplementary Note 3

Supplementally note 2 considers the $3d^6$ electron a free radical since the AC bias in the KPFM measurements unbinds the $3d^6$ electrons from the solid surface, becoming dissolved electrons belonging to water clusters. Without the AC bias at the interface, the $3d^6$ electrons in Fe_3O_4 are fixed by the exchange interactions. As shown in Fig. S6, the spin directions of the $3d^{1\sim5}$ electrons are aligned by the magnetic field, while the $3d^6$ electrons are antiparallel to the $3d^{1\sim5}$ electrons, indicating that the $3d^6$ electron spin is fixed to be antiparallel to the magnetic field. Therefore, when the $3d^6$ electrons are antiparallel to the magnetic field, the energy level of the [$HO_2\cdot\ \ \cdot e^-$] pair is substantially lower than when the $3d^6$ electrons are parallel to the magnetic field. A spin Hamiltonian was constructed to describe this:

$$\mathbf{H_{fix}} = FS_z \quad \text{(S10)}$$

where F is the fix constant. Its value is significantly larger than the thermal energy at 293 K, which is about 25 meV.

Therefore, the spin Hamiltonian of the [HO_2• •e^-] pair can be expressed as follows:

$$\mathbf{H} = \mathbf{H_{ex}} + \mathbf{H_{mag}} + \mathbf{H_{fix}} \tag{S11}$$

Here, it is considered that $J(r) = 0$. Therefore, the spin Hamiltonian can be expressed as follows:

$$\mathbf{H} = g\mu_B BS_z + FS_z = (g\mu_B B + F)S_z \tag{S12}$$

The constant F is much larger than $g\mu_B B$ when the magnetic field B is lower than 0.5 T. Consequently, the effect of the magnetic field on the spin conversion of the [HO_2• •e^-] pair can be disregarded.

Supplementary Note 4

The spin Hamiltonian of the radical pair considering the Zeeman interaction can be expressed as following:

$$H = H_{Zeeman} = g_1\mu_B BS_{z1} + g_2\mu_B BS_{z2} \tag{S13}$$

where g_1 and g_2 are the g factors of the two radicals in the radical pairs.

We concern about the evolution between the singlet spin state and three triplet spin states of the radical pairs, their wave functions are shown below,

$$|S\rangle = (\alpha\beta - \beta\alpha)/\sqrt{2} \tag{S14-1}$$

$$|T_0\rangle = (\alpha\beta + \beta\alpha)/\sqrt{2} \tag{S14-2}$$

$$|T_{+1}\rangle = \alpha\alpha \tag{S14-3}$$

$$|T_{+1}\rangle = \beta\beta \tag{S14-4}$$

The Zeeman energy of these four spin states is calculated as below,

$$E_s = \left\langle(\alpha\beta - \beta\alpha)/\sqrt{2}\middle|g_1\mu_B BS_{z1} + g_2\mu_B BS_{z2}\middle|(\alpha\beta - \beta\alpha)/\sqrt{2}\right\rangle = 0 \tag{S15-1}$$

$$E_{T_0} = \left\langle(\alpha\beta + \beta\alpha)/\sqrt{2}\middle|g_1\mu_B BS_{z1} + g_2\mu_B BS_{z2}\middle|(\alpha\beta + \beta\alpha)/\sqrt{2}\right\rangle = 0 \tag{S15-2}$$

$$E_{T_{+1}} = \langle\alpha\alpha|g_1\mu_B BS_{z1} + g_2\mu_B BS_{z2}|\alpha\alpha\rangle = (g_1 + g_2)\mu_B B \tag{S15-3}$$

$$E_{T_{-1}} = \langle\beta\beta|g_1\mu_B BS_{z1} + g_2\mu_B BS_{z2}|\beta\beta\rangle = -(g_1 + g_2)\mu_B B \tag{S15-4}$$

The energy gap between $|S\rangle$ state and $|T_{\pm 1}\rangle$ states prevents the spin conversion between them, so that only the $S - T_0$ conversion need to be considered in Zeeman interaction.

When we discuss the conversion of two spin states (state S and state T_0) of the radical pair, the wave function of the radical pair can be expressed as a mix state and it evolves with time, as following:

$$\Psi(t) = C_S(t)\varphi_S + C_{T_0}(t)\varphi_{T_0} \tag{S16}$$

And here is the time-dependent Schrodinger's equation:

$$i\hbar\frac{d\Psi(t)}{dt} = i\hbar\left(C_S{}'(t)\varphi_S + C_{T_0}{}'(t)\varphi_{T_0}\right) = H_{Zeeman}(C_S(t)\varphi_S + C_{T_0}(t)\varphi_{T_0}) \tag{S17}$$

Integration of the above equation with $\varphi_S{}^*$ and $\varphi_{T_0}{}^*$ gives the following results:

$$i\hbar C_S{}'(t) = \langle\varphi_S|H_{Zeeman}|\varphi_S\rangle C_S(t) + \langle\varphi_S|H_{Zeeman}|\varphi_{T_0}\rangle C_{T_0}(t) \tag{S18}$$

$$i\hbar C_{T_0}{}'(t) = \langle\varphi_{T_0}|H_{Zeeman}|\varphi_S\rangle C_S(t) + \langle\varphi_{T_0}|H_{Zeeman}|\varphi_{T_0}\rangle C_{T_0}(t) \tag{S19}$$

Here, $\langle\varphi_S|H_{Zeeman}|\varphi_S\rangle = E_S = 0$, $\langle\varphi_{T_0}|H_{Zeeman}|\varphi_{T_0}\rangle = E_{T_0} = 0$, $\langle\varphi_{T_0}|H_{Zeeman}|\varphi_S\rangle = \langle\varphi_S|H_{Zeeman}|\varphi_{T_0}\rangle = \langle(\alpha\beta - \beta\alpha)/\sqrt{2}|g_1\mu_B BS_{z1} + g_2\mu_B BS_{z2}|(\alpha\beta + \beta\alpha)/\sqrt{2}\rangle = (g_1 - g_2)\mu_B B = \Delta g\mu_B B$, and we take $C_S(t) = M_S e^{\rho t}$ and $C_{T_0}(t) = M_{T_0}e^{\rho t}$, we have

$$i\hbar\rho\begin{bmatrix}M_S\\ M_{T_0}\end{bmatrix} = \begin{bmatrix}0 & \Delta g\mu_B B\\ \Delta g\mu_B B & 0\end{bmatrix}\begin{bmatrix}M_S\\ M_{T_0}\end{bmatrix} \tag{S20}$$

In above equation, $i\hbar\rho$ is the eigenvalues of the matrix $\begin{bmatrix}0 & \Delta g\mu_B B\\ \Delta g\mu_B B & 0\end{bmatrix}$, we have

$$\rho = \pm i\left(\frac{\Delta g\mu_B B}{\hbar}\right) = \pm i\omega \tag{S21}$$

$$M_{T_0} = \mp M_S \tag{S22}$$

Thus, $C_S(t)$ and $C_{T_0}(t)$ can be represented as follows

$$C_S(t) = C_1 e^{i\omega t} + C_2 e^{-i\omega t} \tag{S23}$$

$$C_{T_0}(t) = -C_1 e^{i\omega t} + C_2 e^{-i\omega t} \tag{S24}$$

Using $e^{i\omega t} = con\omega t + isin\omega t$ and $e^{-i\omega t} = con\omega t - isin\omega t$, the above two equations become as,

$$C_S(t) = (C_1 + C_2)con\omega t + i(C_1 - C_2)sin\omega t = Acon\omega t + iBsin\omega t \tag{S25}$$

$$C_{T_0}(t) = -Bcon\omega t - iAsin\omega t \tag{S26}$$

Therefore, $C_S(0) = A$ and $C_{T_0}(0) = -B$, and

$$C_S(t) = C_S(0)con\omega t - iC_{T_0}(0)sin\omega t \tag{S27}$$

$$C_{T_0}(t) = C_{T_0}(0)con\omega t - iC_S(0)sin\omega t \quad \text{(S28)}$$

Assuming that the initial spin state of the radical pair is singlet, which implies that $|C_S(0)|^2 = 1$ and $\left|C_{T_0}(0)\right|^2 = 0$, then

$$|C_S(t)|^2 = |C_S(0)con\omega t|^2 = con^2\omega t \quad \text{(S29)}$$

$$\left|C_{T_0}(t)\right|^2 = |-iC_S(0)sin\omega t|^2 = sin^2\omega t = 1 - con^2\omega t \quad \text{(S30)}$$

Equations S29 and S30 suggest that the spin states of the radical pairs convert between singlet state and triplet state at a ω angle frequency, in which $\omega = \frac{\Delta g\mu_B B}{\hbar}$. It can be seen that the S-T spin conversation rate of the radical pairs increases with the external magnetic field.

Supplementary Note 5

A cobalt alloy coated magnetic AFM probe (PPP-MFMR, NanoSensors) was used in the experiments. The magnetic force between the magnetic tip and the Fe_3O_4 sample was detected directly by measuring the force-distance curve without a magnetic field, as shown in Fig. S10a. The force-distance curve implied no significant magnetic force between the magnetic tip and magnetic sample without a magnetic field. However, when a magnetic field was applied, the probe was subjected to a significant magnetic force, even yielding a significant cantilever deflection under the magnetic field (0.5 T), which was observed via the photodetector. Therefore, the amplitude and phase of the probe vibration were significantly affected by the magnetic force, and the measured surface potential using a magnetic tip was far from the actual value, as shown in Fig. S10b (0.5 T). This was why a non-magnetic tip was selected for testing.

Supplementary Note 6

The main scan in our experiments was performed in PeakForce tapping mode, which is a topography scanning mode developed by Bruker. In peakforce tapping mode, the tip contacts the sample surface point by point to record the profilometry, and the force curve is shown in Fig. S11a. The tip approaches to the sample surface until the contact force reaches the set peakforce, and then, the tip is withdrawn from the sample surface. One of the biggest advantages of the PeakForce tapping

mode is the ability to control the contact forces between tip and sample to the level of hundreds pico-newton. In our experiments, the peakforce was set to 300 pN. Such a small contact force cannot lead to significant contact charge transfer between the metallic tip and the samples.

In order to verify that no transferred charge was introduced in PeakForce tapping mode for topography measurement, we use the PeakForce tapping mode to scan the sample surface trying to generate triboelectric charges (with 5 μm scan size, 300 pN peakforce), and then, the surface potential of the scan area is detected in KPFM mode (10 μm scan size). As shown in Figs. S11b, S11c, the results show that no triboelectric charges were introduced in PeakForce tapping mode, no matter in air or in DI water. Moreover, the tip used in experiments was nonmagnetic (SCM-PIT, Pt coated), which was not subjected to magnetic forces. As shown in Figs. S11d, S11e, there is no difference between the force-distance curves of the Pt coated tip on the Fe_3O_4 surface with and without a 0.5 T magnetic field, confirming that the tip is not subjected to magnetic force. Therefore, the enhancement in contact charge could not be from stronger interaction between tip and ferrite when magnetic field is applied.

For the tribovoltaic effect, two materials need to rub against each other to generate enough energy to excite electron-hole pairs. It is unlikely to generate a tribo-current at a slight contact in PeakForce tapping. As shown in Fig. S11f, no tribo-current was detected when the tip scans the Fe_3O_4 surface in PeakForce tapping mode. Therefore, the changes of the charge transfer at DI water and ferrimagnetic solid induced by magnetic field cannot be caused by the tribovoltaic effect.

Supplementary Note 7

The Ampere's force experienced by the Pt-coated AFM cantilever depended on the current through the cantilever (I), the length of the cantilever (L), and the external magnetic field (B), which is expressed as follows:

$$F = IBLsin\alpha \quad \text{(S31)}$$

where α denotes the angle between the current and the magnetic field.

In the DH-KPFM system, the tip and sample electrode could be considered a capacitance, as shown in Fig. S12. The current through the cantilever could be calculated using the following equation:

$$I = \frac{dQ}{dt} = \frac{dCV}{dt} = C\frac{dV}{dt} \tag{S32}$$

where Q denotes the induced charges on the tip and sample surfaces, V denotes the applied AC bias, and C denotes the capacitance between the tip and sample electrode. Equation S32 considered the capacitance of the tip-sample system a constant for convenience since the vibration amplitude of the tip was substantially smaller than the distance between the tip and sample electrode (lift high + thickness of the dielectric film) in the experiments.

The amplitude of the applied AC bias in the experiments was 1 V, and the frequency was 75 kHz, and the AC bias could be expressed as follows:

$$V = sin(2\pi \times 7.5 \times 10^4 t) \tag{S33}$$

The capacitance between the tip and the sample surface was systematically discussed in previous works.[50] In our experiments, the tip radius was less than 20 nm, and the distance between the tip and sample electrode was about 150 nm, so the capacitance between tip and sample electrode was less than 0.1 aF according to the results in Ref. 50. According to Equations S32 and S33, the current through the cantilever could be expressed as follows:

$$I = C\frac{dV}{dt} < 2\pi \times 7.5 \times 10^{-15} cos(2\pi \times 7.5 \times 10^4 t)A \tag{S34}$$

The highest magnetic field in our experiments was 0.5 T, the cantilever length was 225 μm, and the current was perpendicular to the magnetic field. Therefore, the Ampere's force experienced by the Pt-coated AFM cantilever could be expressed as follows:

$$F < 0.5 \times 225 \times 10^{-6} \times 2\pi \times 7.5 \times 10^{-15} cos(2\pi \times 7.5 \times 10^4 t)N$$
$$= 5.29 \times 10^{-18} cos(2\pi \times 7.5 \times 10^4 t)N \tag{S35}$$

Such a small Ampere's force did not contribute to the resonance of the cantilever and could not influence the KPFM contact potential difference result.
